# State Control of Glanders in Minnesota1Read before the American Veterinary Medical Association, New York, September, 1899.

**Published:** 1899-12

**Authors:** M. H. Reynolds

**Affiliations:** St. Anthony Park, Minn.


### THE JOURNAL

OF

### COMPARATIVE MEDICINE AND VETERINARY ARCHIVES.

VOL. XX. *DECEMBER, 1899.* No. 12.

### STATE CONTROL OF GLANDERS IN MINNESOTA.[1]

BY M. H. REYNOLDS, D.V.M.,
ST. ANTHONY PARK, MINN.

### SUMMARY.

Two fundamental considerations: First, protection for the public, including health and property values; second, protection for the owner, which implies the least possible injury or loss consistent with protection of the public. The second consideration frequently overlooked.

Glanders-farcy far more prevalent than commonly realized.

Many cases under natural conditions never develop into clinical glanders. Cases occasionally recover.

Mallein-test practical, but not infallible.

Not always wise to test horses showing marked clinical symptoms.

Horses having glanders-farcy, but showing no clinical symptoms, should be known, recorded, and kept under observation.

All horses which have been seriously exposed in the stable should be tested.

Plan of the Minnesota State Board of Health organization and work.

Extent to which we depend on local health officers.

Minnesota rules, blanks, circulars, etc.; comments.

Results of these methods.

### PROBLEMS.

General problems:
- What constitutes a mallein reaction.
- What horses to kill.

Disposition of horses that react, but show no clinical symptoms.
- Protection of the honest owner.
- Securing prompt information.
- Large cities.
  - Livery stables.
  - Public watering places.

There are two fundamental considerations which must be borne steadily in mind: First, protection for the public, including public

[1] Read before the American Veterinary Medical Association, New York, September, 1899.

health and property values; second, protection for the owner. The latter implies the least possible injury or loss consistent with protection of the public.

I fear that too little attention is paid to the second point. It is undoubtedly possible in most instances to do good work with an outbreak of glanders or hog-cholera or sheep scab, and yet protect the individual owner more than is usually done. The spirit of the law is most important. We can frequently permit an owner to use some of his glandered horses until after seeding or until after harvesting; or the rules may be so worded that an owner may have an opportunity of fattening tuberculous cattle if he cares to do so. Under some circumstances we can permit an owner to sell dressed carcasses of certain hogs, although general quarantine has been ordered upon the place, or we may even permit an owner to sell live hogs out of quarantine under certain conditions. By so doing we not only protect the owner, but we also win his support.

The sanitarian who tries to do his work entirely by rule, and neglects the individual, personal element is very apt to antagonize unnecessarily. He is apt to bring his work or the board with which he is connected into disrepute. He may lose the moral support of veterinarians and stockmen. He is apt to fail in efforts to push any movement that needs the support of stockmen. It is occasionally necessary to force issues, but we always make it a point to treat the owner as fairly and generously as possible, and so manage the case as to win his confidence and support. The veterinary sanitarian who does not have the support of his prominent stockman will fail. Legal phrases do not constitute a law.

It is becoming evident that glanders-farcy is far more prevalent than we have realized. So long as veterinarians diagnosed on external symptoms, or tested only horses that showed evident symptoms of glanders-farcy, this state of things did not become apparent. It is no rare experience to test a stable of ten or fifteen horses and have a large number react—many of them previously unsuspected. When we post-mortem such horses it becomes evident that mallein has told us the truth, and we are forced to believe that glanders is much more prevalent than we had realized.

There are undoubtedly many cases which, under natural conditions, would never develop into clinical glanders, but that does not alter our responsibility with them or lessen the danger from those that become infectious. Grant that some cases of glanders recover, but this does not alter the fact that there is also a large number that do not recover, but, on the contrary, develop into advanced and very infectious cases. I would not overlook or underestimate the fact that glanders cases may make complete recoveries, and we must not forget to give the owner's account credit for this possibility. On the other hand, we must not be blind to probable dangers. I think that our rules concerning glanders-farcy take this into account and give the owner a safe credit.

There can no longer be reasonable doubt as to the practical reliability of mallein as a diagnostic; but we occasionally have cases where the diagnosis is very evident, and yet the horse gives an indefinite reaction, or even fails to react at all. Fortunately, such animals usually show evident symptoms of glanders and a diagnosis on clinical evidence can be made. This suggests the thought that it is not always wise to test horses which show clinical symptoms. We may have reasonable evidence that a horse is affected with glanders-farcy, but if we test such a horse and he fails to react, we have brought an unjust discredit upon our work and the mallein test. There is nothing to be gained by testing a plain case of glanders.

The next proposition is, that we ought to know where the mild and latent cases are, and keep such cases under observation even though it be true that comparatively few develop into clinical cases. For this reason I believe that it is wise to test all horses which have been seriously exposed in the stable or by long association in the open air. A rule of this sort is difficult to enforce, and the work must be carefully managed or it causes owners great inconvenience, possibly great financial loss, and it may arouse very violent opposition. It is the usual custom for State veterinarians and similar officials to test only horses which show clinical symptoms of glanders-farcy, or individual cases in which there is doubt concerning the diagnosis. I have already expressed the opinion that there is nothing to be gained, but frequently something to lose, by testing plain cases of glanders-farcy, and I fully believe that these gentlemen are testing the wrong horses. It is our common experience that when we test a stable of horses which has included for two weeks or longer a clinical case of glanders-farcy we find one or more unsuspected horses which react. I know that this is true in Minnesota, and I suspect that it is true elsewhere.

Two years ago I sent a circular letter to prominent veterinarians in the United States, and especially to those engaged in this kind of work, asking for an expression of opinion on several questions, including the following:

Do you test all horses that have been exposed to infection in the stable with glandered horses?

Do you feel justified in ordering the destruction of all horses which react?

I received a quite generous response. Three of my correspondents suggested the desirability of testing all horses that had been exposed in the stable, but only one was actually doing so. Several of the most prominent veterinarians in the United States reported that they were using mallein only for the purpose of testing horses which showed evident symptoms of glanders and for the purpose of deciding doubtful diagnoses. The majority of my correspondents were not in favor of destroying horses that showed no evidence of glanders beyond a mallein reaction. Only one veterinarian replied that all horses reacting on mallein test should be destroyed at once.

I have repeatedly submitted to Minnesota veterinarians at State Association meetings and in private conversation the question as to whether they believe it wise to test all horses that have been exposed, and with very few exceptions the answer has been "Yes." So I take it that this plan has not been a failure for Minnesota.

My reasons for taking this position several years ago were as follows: A serious percentage of horses exposed in the stable become infected. We have no evidence but the mallein-test that will enable us to detect such cases. We cannot under ordinary conditions know whether such horses are or are not infectious. We cannot know under ordinary conditions which will develop into clinical cases.

*Minnesota Organization:* I have previously discussed before this body the organization of the Minnesota State Board of Health and something of its methods. I wish to present some of our rules, circulars, etc., in this connection, and in order that they may be understood I would ask you to bear in mind that all police authority for dealing with infectious diseases of domestic animals is given to the State and local boards of health. The State Board of Health consists of eight physicians and one veterinarian. The work is divided into three departments: that of the secretary and general executive officer, bacteriological department, and veterinary department. The Experiment Station veterinarian is a member of the State Board of Health and Director of the Veterinary Department, with authority and duties resembling those usually given to a State veterinarian. All our cities, towns, and villages have local health officers. Every board of township supervisors is also a local board of health and has considerable authority. The Minnesota organization, then, consists of the State Board of Health with its three departments and approximately eighteen hundred and forty local boards of health.

### Minnesota State Board of Health.

### Rules and Suggestions Concerning Glanders–Farcy.

*Rules.* In all ordinary cases of suspected glanders-farcy, first quarantine the suspected animals, then call a competent veterinarian, who should make such examination and tests as he may deem necessary. The further action of the board to be largely determined by the diagnosis and advice of the veterinarian.

All horses must be included in this preliminary quarantine that are discharging from the nose or that have had recent sores upon the body, and all horses that have worked as mates with such horses.

Test with mallein all horses that have been exposed in the stable to cases that may be reasonably suspected of having glanders-farcy.

The following must be destroyed without delay: All horses that have given one clear reaction on mallein-test and show any external symptoms of the disease, or any chronic lung-trouble, such as chronic cough or thick wind; all horses which show positive symptoms of the disease without a clear mallein reaction.

Horses which have reacted but show no other symptoms of the disease may either be killed at once or continued under quarantine for retest for a period of not less than thirty days or more than one year from date of first test. These horses must be retested at the end of the quarantine period, and shall not be released from quarantine unless they fail to react on retest. If at any time within the year these horses show external symptoms of the disease they must be killed.

Quarantine may not be released in any case until the owner has disinfected the premises as directed by health-officers.

In all cases where retests are made the second dose must be one-half larger than the first.

Carcasses must be destroyed by burning if practical, otherwise buried under four feet of earth.

*Violation of Quarantine Defined.* It shall be deemed a violation of quarantine for any person to knowingly remove, authorize or cause to be removed, any horse or mule quarantined on account of glanders-farcy from the farm whereon it is quarantined.

It shall be deemed a violation of quarantine for any person to knowingly cause, authorize or permit to be placed, any horse or mule, except those legally quarantined, in any stable or enclosure that is under quarantine on account of glanders-farcy.

*An Act to Prevent the Spread of Contagious and Infectious Diseases Among Domestic Animals in this State.* (Act of 1897.)

Section 9. Any person violating any provision of this act, or any rule or regulation made by the State Board of Health, or by any local board of health, or any order made by any such board under the authority hereof, shall be guilty of a misdemeanor and be punished by a fine of not less than twenty-five (25) or more than one hundred (100) dollars, or by imprisonment for not less than thirty (30) or more than ninety (90) days.

*Suggestions.* The mallein-test should be conducted as nearly as possible according to the plan given in the blanks sent out from this office. This plan for the test has been found very convenient, and it is important for the proper keeping of office records that the injection be made and the temperature taken at uniform periods by different veterinarians.

It is most economical and usually most satisfactory to have the veterinarian who makes the examination and conducts the mallein-test to finish the work at one trip.

Domestic animals affected with any infectious disease are considered by law as having no commercial value so far as the work of boards of health is concerned.

Health officers should realize that the law imposes important duties upon them and severe penalty for neglect or refusal to perform these duties. They should impress the fact upon the owners that the law also imposes duties and penalties upon them.

Glanders-farcy is very fatal to human beings and easily communicable to them by inoculation through cuts or scratches upon the hands or face, or if the matter gets into the nose or eyes.

Comments: Please note that all horses which have been exposed in the stable to cases which may be reasonably suspected of having glanders-farcy must be tested. All horses that have given one clear reaction and show any clinical symptoms of the disease, and all horses which show positive symptoms of the disease with or without mallein-reaction, must be destroyed.

Horses which react but show no external symptoms may be killed or continued under partial quarantine at the option of the local or State Board of Health. Horses which develop symptoms of glanders during the period of quarantine must be destroyed.

(To be continued.)

